# Educating the masses: suggestions for improving online concussion information via the mainstream media

**DOI:** 10.2217/cnc-2016-0026

**Published:** 2016-12-15

**Authors:** Osman Hassan Ahmed, Tracy Blake, Eric E Hall

**Affiliations:** 1 Faculty of Health & Social Sciences, Bournemouth University, Bournemouth, UK; 2 The FA Centre for Disability Football Research, The Football Association, St George's Park, Burton-Upon Trent, UK; 3 Sport Injury Prevention Research Centre, University of Calgary, Calgary, Canada; 4 Department of Exercise Science, Elon University, NC 27244, USA

**Keywords:** concussion, mTBI, recovery

It was said by Jim Morrison that “whoever controls the media controls the mind” [1] and the power of the media to shape and inform opinions has never been greater. Mainstream news outlets play a pivotal role in the modern age, and public health is included in the scope of influence of the media [2]. Previous research has explored how social media has portrayed sports concussion; Twitter has been highlighted for its rapid dissemination mechanisms [3], while concussion-related content on YouTube predominantly arose from news and media organizations [4]. Given the current high profile of sports concussion in the news, it is unsurprising that attention has turned toward its representation and portrayal in the media.

A recent study from Ahmed and Hall [5] discussed the description of sports concussion in online news articles, and demonstrated inconsistencies in the terminology used to describe concussion. From 200 news articles retrieved, the terms ‘head injury’ and ‘brain injury’ were only used in 30 and 21% of cases, respectively, suggesting that the seriousness of these injuries may be downplayed by the terminology used to describe them. In addition, 10% of the articles used inappropriate modifiers, that is, words such as ‘mild’ or ‘moderate’ to describe the concussive injury. These descriptions were often made by journalists, leading the authors to create a checklist (the ‘Media Concussion Checklist’) in order to educate journalists and facilitate consistency in reporting about concussions.

The descriptions used by the media in reference to concussion-related decision-making are noteworthy; for example, the decision by a 2016 Olympic Games gymnast to continue competing after a head injury being described as ‘lionhearted’ [6]. This phrasing creates a link between an individual's personal character and healthcare decision-making that is inherently problematic. Although this scenario generated a debate regarding the logic of their decision to continue competing [7], this form of value-based description of concussion decision-making opens the doors for the opposite decision (i.e., removal from play) to be construed negatively (e.g., using descriptors such as ‘mouselike’ or ‘cowardly’).

This is especially true given that decision-making processes surrounding injury disclosure and return to play are complex and multifactorial. Symptom-reporting remains a cornerstone of current concussion evaluation practice standards, however studies have demonstrated that athletes are not always consistent in disclosing potential concussive injuries [8]. The role that masculinity plays in association with concussion reporting has also been explored; Anderson and Kian [9] have suggested that there is a historical underpinning of masculinity to concussion reporting with regard to football in the USA, and similar cultural underpinnings are discussed by Hokowhitu toward Maori rugby players in New Zealand [10]. Although the mainstream media may not be able to overcome reporting and cultural issues by themselves, their description of these incidents may help to shape the public's perception of sports concussion as a whole.

The importance of the media is especially pertinent as the knowledge of the general public toward concussion has been shown to be variable at best [11], and thus it is not unreasonable to expect the journalistic community to show a degree of responsibility toward discussing this injury. This process of transferring information (‘knowledge transfer’ or KT) has been discussed in depth by Provvidenza *et al*. with respect to sports concussion [12]. In order to better facilitate the KT process it is clear that greater cohesion between the medical and media communities is needed. The media concussion checklist is a preliminary step in the process of helping to guide members of the media in their production of news items that both tell the story and align with the scientific evidence and best-practice guidelines.

The pursuit toward more accurate reporting of medical conditions is not unique to sports concussion, and suggestions for journalists reporting on eating disorders are already in existence [13]. A hallmark of an open and equitable society is for an unimpeded media, which can freely report on events, including sports injuries. Penalties for inaccurate reporting and ‘outing’ of journalists who use incorrect terminology to describe concussion are not likely to assist the KT process, or ultimately benefit athletes and players. It stands to reason therefore that the medical community should not attempt to shackle the journalistic world; rather they should work symbiotically with journalists, to allow them to create more medically accurate content for their readers. This will also assist the medical community and concussion researchers to achieve a wider reach with correct concussion management messages, especially if this is written in a user-friendly format.

Although there has been focus on concussion education for athletes [14], parents [15], coaches [16] and healthcare professionals [17], to date there has not been an input toward the knowledge levels of sports journalists toward concussion. Given that sports journalists are unlikely to have had any formalized medical training, this constitutes a knowledge gap that could be positively addressed. It is unrealistic and indeed unfeasible to expect all sports journalists to complete a dedicated medical module; however, the routine and regular inclusion of journalists in the provision of education materials such as the ‘HEADS UP to Youth Sports’ [18] and invitations to symposiums/sports medicine conferences where concussion is addressed would be a proactive step to remedy this.

Creating a culture of journalists who are informed and educated about concussion means that they would be able to pick up on incorrect terminology during these interviews, and edit their reporting appropriately. In association with education, another consideration is the importance of verbatim and nonverbatim quotes used in news stories. If the verbatim quotes from athletes and coaches include inappropriate descriptors of concussions (e.g., “he just had a ding”) or modifiers of concussions (e.g., “I just got a little concussion”), then journalists could consider the use of nonverbatim quotes to more accurately inform their readers about this injury. A recent article discussed the importance of using nonverbatim quotes to protect anonymity on information gathered from discussion boards [19], and this concept could be expanded to journalists in their reporting of concussion.

In addition to 2016 being an Olympic/Paralympic year, it was also the year when the 5th International Consensus Conference on Concussion in Sport was held in Berlin, Germany. These consensus conferences are the pinnacle of research related to sports concussion, and help to drive a lot of the thinking and evolution in the field. The 2016 conference reinforced previous sentiment that there are still lots of unknowns related to sports concussion [20], and the next 4 years will hopefully see an increase in the medical community's knowledge on concussion. It is not idealistic to hope that in parallel with this there will also be an increase in the knowledge of the media in relation to concussion, which will create a more nuanced and educated sports media, and in turn be of benefit to athletes and the wider community.
